# Symptom Reduction and Deprescribing in a Patient With Treatment-Resistant Depression Using Sublingual Ketamine Troches: A Case Report

**DOI:** 10.7759/cureus.87343

**Published:** 2025-07-05

**Authors:** Elizabeth Francis

**Affiliations:** 1 School of Nursing, Duke University, Durham, USA

**Keywords:** complex-ptsd, ketamine infusion, low dose ketamine, major depressive disorder (mdd), psychedelic-assisted therapy, psychiatry and mental health, treatment resistant depression (trd)

## Abstract

Treatment-resistant depression (TRD) remains a significant clinical challenge, often accompanied by polypharmacy and comorbid anxiety or trauma-related disorders. This report describes a 37-year-old male with longstanding major depressive disorder (MDD), post-traumatic stress disorder (PTSD), and social anxiety disorder who demonstrated limited response to traditional pharmacologic strategies, including escitalopram, quetiapine, trazodone, and gabapentin. After initiating low-dose sublingual ketamine troches, the patient experienced clinically meaningful improvements in depression and anxiety scores. These improvements were accompanied by successful tapering and discontinuation of four psychotropic medications. No adverse effects were reported. This case highlights the potential utility of sublingual ketamine in reducing symptom burden and supporting deprescribing efforts in complex TRD presentations.

## Introduction

Treatment-resistant depression (TRD) affects a significant portion of patients with major depressive disorder, often requiring multi-drug regimens that may lead to sedation, cognitive impairment, and poor adherence [1,2]. The U.S. Food and Drug Administration (FDA) and the European Medicines Agency (EMA) define TRD as the failure to respond adequately to at least two different antidepressant treatments, assuming that the treatments were appropriate in terms of dosage and duration, and that the patient followed the prescribed regimen [3]. It's currently believed that over 30% of individuals with depression fall into this category [3]. However, a notable portion of these cases may be classified as pseudo-resistant, often due to factors like inadequate treatment attempts or poor adherence [3]. 

Ketamine, a glutamatergic modulator, has demonstrated rapid antidepressant effects and is now being explored in various administration routes beyond intravenous infusion [4,5]. Ketamine is a non-competitive N-methyl-D-aspartate (NMDA) receptor antagonist with rapid-acting antidepressant and analgesic properties [6]. Sublingual administration offers moderate bioavailability (~20-30%) by partially bypassing first-pass metabolism, resulting in onset of effects within 15-30 minutes and prolonged duration compared to oral dosing [7]. Ketamine is metabolized hepatically via cytochrome P450 to norketamine, an active metabolite contributing to its therapeutic effects [4,5]. The sublingual route provides a convenient, non-invasive option for outpatient treatment of conditions like treatment-resistant depression and chronic pain, though variable absorption requires careful dosing and monitoring. This case report presents a case of successful treatment of TRD with sublingual ketamine troches, resulting in both symptom improvement and reduction of polypharmacy.

## Case presentation

A 37-year-old male presented to a psychiatric clinic in January 2025 with longstanding symptoms of major depressive disorder (MDD), post-traumatic stress disorder (PTSD), and social anxiety. He described persistent low mood, emotional numbness, intrusive memories, withdrawal from social situations, poor concentration, disrupted sleep, and reduced capacity for pleasure. He also reported experiencing passive suicidal ideation several times per week. His family psychiatric history includes generalized anxiety disorder (GAD) and MDD in both parents. He is employed as a sales trainer at a Fortune 500 company. He has no significant medical conditions, and lab results from his primary care provider three months prior were within normal limits. His extended family medical history is unknown.

Over the previous 10 years, he had undergone multiple medication trials, each at therapeutic doses and durations, with limited effectiveness or problematic side effects. These trials included bupropion XL 300 mg daily, buspirone 20 mg twice daily, venlafaxine 150 mg daily, citalopram 20 mg daily, hydroxyzine 50 mg twice daily, and Adderall 10 mg daily (Table 1). Each was trialed for at least two months. Although he engaged in biweekly psychotherapy with moderate benefit, pharmacological response remained minimal.

**Table 1:** Summary of Previous Pharmacologic Trials NDRI: norepinephrine-dopamine reuptake inhibitor, SNRI: serotonin-norepinephrine reuptake inhibitor

Medication	Dosage	Class/Indication	Treatment Outcome
Bupropion XL (Wellbutrin)	300 mg daily	NDRI Antidepressant	Minimal mood improvement; depressive symptoms persisted
Buspirone	20 mg twice daily	Anxiolytic (5-HT1A partial agonist)	Limited reduction in anxiety symptoms, side effects (dizziness, headaches)
Venlafaxine (Effexor XR)	150 mg daily	SNRI Antidepressant	Inadequate response; mood and anxiety symptoms persisted
Hydroxyzine	50 mg daily	Antihistamine (used for anxiety)	Continued anxiety despite regular use
Adderall (Mixed Amphetamines)	10 mg daily	Stimulant (augmenting strategy)	No meaningful benefit in mood or overall functioning

Despite these efforts, he continued to experience moderate to severe depressive and anxiety symptoms, meeting criteria for TRD.

At the time of intake, the patient's medication regimen for the last two years included escitalopram 20 mg daily, quetiapine 12.5 mg three times daily, quetiapine 100 mg nightly, trazodone 50 mg nightly, and gabapentin 100 mg twice daily (see Table 2). His baseline symptom severity was notable, with the nine-item Patient Health Questionnaire (PHQ-9) score of 23 indicating severe depression, a Generalized Anxiety Disorder (GAD-7) score of 19 indicating severe anxiety, and a Montgomery Asberg Depression Rating Scale (MADRS) score of 33, consistent with moderate to severe depression [8-10]. These symptoms were impacting his life in a variety of ways, which included reduced concentration at work, withdrawal from social functions due to anxiety and fear, and difficulty maintaining hygiene. 

**Table 2:** Current Medication Regimen SSRI: selective serotonin reuptake inhibitor

Medication	Dosage	Class/Indication	Treatment Outcome
Escitalopram (Lexapro)	20 mg daily	SSRI Antidepressant	Partial response; core depressive symptoms persist
Quetiapine (Seroquel)	12.5 mg three times daily	Atypical Antipsychotic (anxiolytic use)	Inadequate relief of anxiety and depressive symptoms
Quetiapine (Seroquel)	100 mg nightly	Atypical Antipsychotic (sedative/adjunct)	No sustained mood stabilization
Trazodone	50 mg nightly	Serotonin Antagonist/Reuptake Inhibitor	Partial response for sleep
Gabapentin	100 mg twice daily	Anticonvulsant (off-label for anxiety)	Limited benefit; anxiety symptoms ongoing

Given his history of poor response to conventional medications and ongoing functional impairment, a decision was made to initiate ketamine. Due to limited insurance coverage for Spravato, the troches were prescribed three nights per week for the first two weeks, then increased to four and eventually five nights weekly. His current medications were continued during induction. He was monitored weekly for the first month and then biweekly; vital signs remained stable. He denied any adverse effects throughout the ketamine treatment.

**Figure 1:**
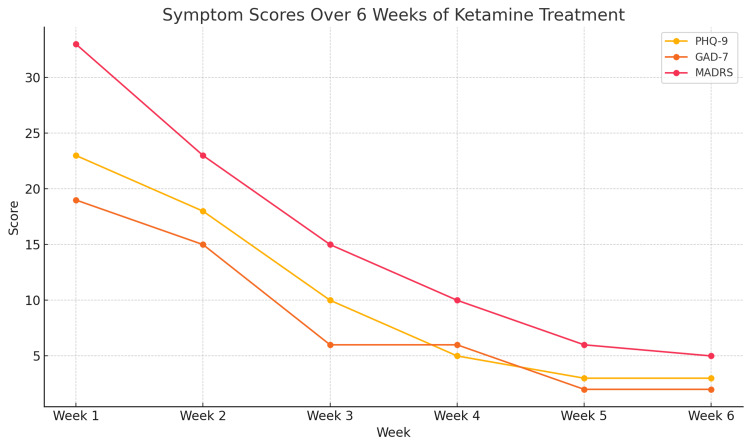
Symptom Scores Over Six Weeks of Ketamine Treatment PHQ-9: Patient Health Questionnaire-9; GAD-7: Generalized Anxiety Disorder-7; MADRS: Montgomery Asberg Depression Rating Scale

Following three weeks of ketamine treatment, the patient experienced a significant reduction in symptom severity. His Patient Health Questionnaire PHQ-9 score dropped to 3, consistent with minimal to no depressive symptoms; his GAD-7 score decreased to 2, indicating minimal to no anxiety; and his MADRS score reduced to 5, reflecting minimal depressive symptoms (see Figure 1). Subjectively, he reported improved mood, normalized sleep, re-engagement in social activities, reduced hypervigilance, and resolution of suicidal thoughts. Mental status examinations over the course of treatment demonstrated marked improvement in the patient’s overall presentation. Initially characterized by a constricted affect, distractible attention, and an anxious mood, his mental status gradually shifted to a more congruent affect, sustained attention, and a euthymic mood. These changes were accompanied by noticeable increases in energy, cognitive clarity, and day-to-day functioning. The patient also reported a significant enhancement in his overall quality of life as treatment progressed.

After the first month of ketamine treatment, the patient began a gradual discontinuation of his existing medications, starting with a hyperbolic taper of escitalopram, followed sequentially by trazodone, gabapentin, and the daytime doses of quetiapine. His nighttime quetiapine dose was reduced from 100 mg to 50 mg. The tapering process was well tolerated, with no signs of withdrawal or mood destabilization. By the five-month follow-up, he remained in stable remission while continuing sublingual ketamine five nights per week, a reduced dose of nighttime quetiapine (50 mg), and biweekly psychotherapy.

## Discussion

This case illustrates the clinical trajectory of a patient with TRD who had previously undergone multiple medication trials, including bupropion, venlafaxine, buspirone, hydroxyzine, and stimulants, without meaningful response. His presentation is typical of many TRD cases, where cumulative pharmacologic burden often fails to resolve core affective and anxiety symptoms [1,2].

Sublingual ketamine was selected based on emerging clinical data supporting its feasibility in outpatient practice and its growing utilization in mood disorders [4-7]. After six weeks of structured treatment, the patient exhibited substantial reductions in validated symptom scores and was able to safely discontinue four concurrent psychotropic medications. These findings support the therapeutic potential of ketamine in both symptom control and medication streamlining for patients with complex psychiatric profiles.

Preliminary evidence suggests sublingual ketamine may be effective for depression and anxiety. In a retrospective analysis, approximately 50% of patients experienced clinical improvement after three sessions [5]. A prospective telehealth study using sublingual ketamine (300-450 mg) reported a 60% response rate after four weeks [6]. Other studies have also found improvements in mood, cognition, and sleep [4,7].

Sublingual ketamine has an estimated bioavailability of 20-30%, bypassing much of hepatic first-pass metabolism, which contributes to its faster onset and therapeutic action [11]. Long-term use has been shown to be tolerable in chronic pain settings, with durations ranging from two to 89 months [11]. Common side effects include mild dizziness, nausea, and lightheadedness, though these are generally transient [5-7]. The risk of abuse is considered low at therapeutic doses, especially in monitored settings [11].

Recent trials of oral ketamine tablets further confirm antidepressant efficacy with minimal sedation or cardiovascular effects, expanding the potential role of non-parenteral ketamine formulations [11-14]. 

Although randomized controlled trials are limited, real-world data suggest sublingual ketamine may offer a safe and effective option for TRD patients seeking alternatives to intravenous or intranasal routes. Ketamine therapy is used in individuals 18 years and older, and can be contraindicated in certain populations, such as those with uncontrolled hypertension and active substance misuse. Thorough patient history including medication trials, doses, durations, substance use history, and medical history should be done prior to initiation. Regular monitoring should be done on blood pressure, for side effects, and symptom relief on validated screening tools. This case highlights both symptom relief and safe deprescribing, an increasingly important outcome in polypharmacy-laden psychiatric care.

## Conclusions

Sublingual ketamine troches present a promising therapeutic option for individuals with treatment-resistant depression and PTSD, particularly those with comorbid conditions. In this case, ketamine was associated with meaningful symptom improvement and enabled the successful reduction of multiple psychotropic medications. Its sublingual form offers a more accessible and less invasive alternative to intravenous or intranasal routes, with potential for use in outpatient settings. While early results are encouraging, further research is needed to better understand its long-term safety, risk of misuse, optimal dosing strategies, and integration into clinical practice. Current substance use and blood pressure should be closely monitored throughout treatment. Continued investigation will help clarify ketamine’s role in reducing medication burden and improving outcomes in complex, treatment-resistant cases.
